# Cost-effectiveness of MRI compared to mammography for breast cancer screening in a high risk population

**DOI:** 10.1186/1472-6963-9-9

**Published:** 2009-01-13

**Authors:** Susan G Moore, Pareen J Shenoy, Laura Fanucchi, John W Tumeh, Christopher R Flowers

**Affiliations:** 1 Department of Hematology and Oncology, School of Medicine, Winship Cancer Institute, Emory University, Atlanta, USA; 2 School of Medicine, University of Virginia, Charlottesville, Virginia, USA

## Abstract

**Background:**

Breast magnetic resonance imaging (MRI) is a sensitive method of breast imaging virtually uninfluenced by breast density. Because of the improved sensitivity, breast MRI is increasingly being used for detection of breast cancer among high risk young women. However, the specificity of breast MRI is variable and costs are high. The purpose of this study was to determine if breast MRI is a cost-effective approach for the detection of breast cancer among young women at high risk.

**Methods:**

A Markov model was created to compare annual breast cancer screening over 25 years with either breast MRI or mammography among young women at high risk. Data from published studies provided probabilities for the model including sensitivity and specificity of each screening strategy. Costs were based on Medicare reimbursement rates for hospital and physician services while medication costs were obtained from the Federal Supply Scale. Utilities from the literature were applied to each health outcome in the model including a disutility for the temporary health state following breast biopsy for a false positive test result. All costs and benefits were discounted at 5% per year. The analysis was performed from the payer perspective with results reported in 2006 U.S. dollars. Univariate and probabilistic sensitivity analyses addressed uncertainty in all model parameters.

**Results:**

Breast MRI provided 14.1 discounted quality-adjusted life-years (QALYs) at a discounted cost of $18,167 while mammography provided 14.0 QALYs at a cost of $4,760 over 25 years of screening. The incremental cost-effectiveness ratio of breast MRI compared to mammography was $179,599/QALY. In univariate analysis, breast MRI screening became < $50,000/QALY when the cost of the MRI was < $315. In the probabilistic sensitivity analysis, MRI screening produced a net health benefit of -0.202 QALYs (95% central range: -0.767 QALYs to +0.439 QALYs) compared to mammography at a willingness-to-pay threshold of $50,000/QALY. Breast MRI screening was superior in 0%, < $50,000/QALY in 22%, > $50,000/QALY in 34%, and inferior in 44% of trials.

**Conclusion:**

Although breast MRI may provide health benefits when compared to mammographic screening for some high risk women, it does not appear to be cost-effective even at willingness to pay thresholds above $120,000/QALY.

## Background

In the United States, one in eight women will be diagnosed with breast cancer during her lifetime [1]. In 2008, an estimated 182,460 cases of breast cancer will occur, accounting for 26% of all cancer cases in women [1] Current consensus screening recommendations divide women into normal and high-risk categories after using physical examination and clinical judgment as a starting point [2]. According to the National Comprehensive Cancer Network (NCCN) Clinical Practice Guidelines [2,3] women at increased risk of breast cancer include those with (i) a history of thoracic or mantle irradiation, (ii) a strong family history or genetic predisposition, (iii) lobular carcinoma in situ or atypical hyperplasia, (iv) a prior history of breast cancer, and/or (v) those over 35 years of age with a 5-year risk of invasive breast cancer ≥ 1.7% according to the modified Gail Model. This model calculates risk based on current age, age at menarche, age at first live birth, nulliparity, previous breast biopsies, atypical hyperplasia, and race, though it has not been conclusively validated in non-Caucasian women [2]. The 5-year risk of ≥ 1.7% is the average risk of a women at the median age of breast cancer diagnosis in the United States [4]. Women with a strong family history or genetic disposition are defined as those with BRCA1/BRCA2 mutations, or a personal family history of breast cancer and one of several other familial risk categories, including being diagnosed before age 40, or before age 50 with one or more close blood relative with breast cancer, or a close family member meeting any of the other criteria [4]. It has been estimated that the risk of developing breast cancer in those with BRCA1 or BRCA2 mutations is 45% to 65% respectively [4].

The screening algorithms for women at increased risk are based on the five aforementioned categories. For women under 25 years of age with a strong family history or genetic predisposition, the recommendation is for annual clinical breast examinations and regular breast self-examination starting at age 18 years [5-7]. The NCCN screening recommendations for women ≥ 25 years in this risk category include annual mammogram and breast MRI screening starting at age 25, or based on earliest age of onset in the family, consideration of prophylactic mastectomy, consideration of chemoprevention options, and consideration of investigational imaging and screening studies [8]. Screening mammography has been shown to reduce mortality from breast cancer by approximately 24% in women between the ages of 50 and 70 [2]. One modelling study found that screening decreased the mortality from breast cancer by 7% – 23%, and that when combined with adjuvant therapy, the rate declined by 25% – 38% [9-12]. Despite some conflicting evidence, screening recommendations endorse annual mammography in normal risk women starting at age 40 years [9-12]. In high-risk women, who tend to develop breast cancer at earlier ages, however, mammography screening is less sensitive, largely due to problems detecting cancer in dense breast tissue. In several studies of high-risk women, including those with BRCA1 and BRCA2 mutations, yearly screening mammography had sensitivities ranging from 25% to 36% [13]. Furthermore, observational studies of BRCA mutation carriers suggest that 50% of breast cancers in this population present between screening mammograms [4,7].

MRI is not affected by breast density, and the recent inclusion of breast MRI in the screening guidelines is based on studies suggesting that high-risk women may benefit from MRI screening [14]. Several studies have reported the sensitivity of MRI screening in high-risk women to be between 77% and 91% [14]. Screening with MRI has also been shown to detect breast cancer in earlier stages in high-risk women [15]. Unfortunately, spontaneous hormone-induced enhancement may occur, leading to false positive test results and unnecessary biopsies in women screened by MRI over mammography. Accordingly, MRI has lower specificity of 90% as compared to 95% for mammography [13].

Though MRI is more sensitive than mammography in a high-risk population, it has not yet been shown to reduce mortality [16]. MRI is also approximately 10 times more expensive than mammography and, due to the comparatively lower specificity, leads to increased costs in the form of potentially unnecessary diagnostic examinations, biopsies, and anxiety [11]. Although, based on existing evidence, current screening guidelines recommend consideration of MRI screening in this high-risk population [11], its use remains controversial.

Cost-effectiveness analysis, however, can play an important role to help determine the role of MRI in screening women at high-risk for breast cancer. The objective of this study is to determine the cost-effectiveness of MRI in screening women with a ≥ 15% cumulative lifetime risk of breast cancer by using a Markov decision model in a hypothetical cohort of patients.

## Methods

### Decision Model

**Figure 1:**
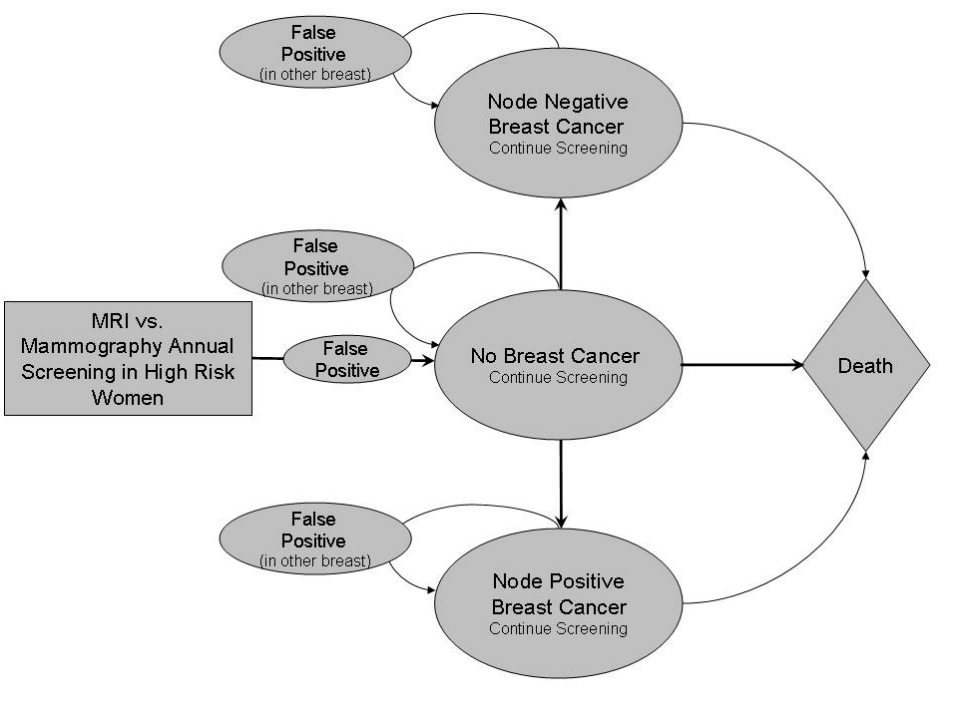
**The Markov model**. This model considers one cycle to be a full year, accounting for each time a patient underwent screening. Note that each breast is tracked independently, but patient state is determined by occurrence or no occurrence of cancer in the first breast.

We developed a Markov decision model using a hypothetical cohort of patients to compare annual breast cancer screening over 25 years with either breast MRI or mammography among young women with ≥ 15% cumulative lifetime risk of breast cancer according to the Claus tables. The Claus tables is a breast cancer risk assessment tool that estimates risk based on maternal and paternal family history, 1st and 2nd degree relatives, age, and family history of ovarian cancer. The main limitation of this method, however, is that it does not incorporate risk factors other than family history. The Markov model considered one cycle to be a full year, accounting for each time a patient underwent screening. In the model, patients are initially screened with either MRI or mammography. The results of the diagnostic exam can either show no breast cancer, node negative breast cancer, or node positive breast cancer. In all situations, the patients continue annual screening until death. Probabilities of living differ for the three scenarios and, like all other model probabilities including sensitivity and specificity of each screening strategy, are based on published literature. The structure of the model is shown in Figure 1.

Probabilities of MRI and mammography test results and true positives were distinguished based on BI-RAD scores, where BI-RADS 0 = "need additional imaging," BI-RADS 3 = "probably benign finding," BI-RADS 4 = "suspicious abnormality", and BI-RADS 5 = "highly suggestive of malignancy". Probability ranges were obtained by constructing 95% confidence intervals for proportions derived from the literature using normal approximations to the binomial distribution. The probabilities used in the model and ranges explored in univariate sensitivity analyses are shown in Table 1[11,17-22].

**Table 1:** Probabilities used in the model

**PROBABILITIES**	**MRI**		**Mammography**	
	**Base Case**	**Range**	**Base Case**	**Range**
Positive[11]	0.108	0.047–0.169	0.054	0.010–0.098
BI-RADS 0/3[11]	0.856	0.787–0.925	0.898	0.838–0.957
BI-RADS 4/5[11]	0.144	0.075–0.213	0.102	0.043–0.162
True Positive BI-RADS 0/3[11]	0.028	0.000–0.061	0.035	0.000–0.070
False Positive BI-RADS 0/3[11]	0.972	0.939–1.000	0.965	0.930–1.000
Node Positive[11]	0.214	0.134–0.295	0.564	0.467–0.661
Node Negative[17,18]	0.786	0.705–0.866	0.436	0.339–0.533
True Positive BI-RADS 4/5[17,18]	0.323	0.231–0.415	0.478	0.380–0.576
False Positive BI-RADS 4/5[17,18]	0.677	0.585–0.769	0.522	0.424–0.620
False Negative Node Positive[19]	1.000	0.700–1.000	1.000	0.700–1.000
Negative[20]	0.892	0.831–0.953	0.946	0.902–0.990
True Negative[21]	0.997	0.985–1.000	0.993	0.977–1.000
False Negative[21]	0.004	0.000–0.015	0.007	0.000–0.023
Live Node Positive[22]	0.970	0.937–1.000	0.970	0.937–1.000
Live Node Negative	0.990	0.970–1.000	0.990	0.970–1.000
Live no cancer	0.998	0.989–1.000	0.998	0.989–1.000

Abbreviations: BI-RADS, Breast Imaging Reporting and Data System classification

### Costs

Model costs for physician, hospital and laboratory services were based on methodology described in previously published work [11], using Centers for Medicare & Medicaid Services reimbursement data to estimate costs unadjusted for geographic location and therefore representing a national average. Medication costs were obtained from the Federal Supply Scale (FSS). Costs of care and ranges explored in sensitivity analyses are shown in Table 2 (Additional File 1-Current Procedural Terminology codes).

**Table 2:** Costs used in the model.

**PROCEDURES**	**COST ($)**	**RANGE ($)**
Local Therapy (Node negative) – Pre-op Evaluation, Lumpectomy with SN biopsy, Lumpectomy Re-excision, WBRT-B post lumpectomy (Konski), Mastectomy with SN biopsy, Breast Reconstruction	12,623.41	8,387.27 – 19,405.81

Local Therapy (Node positive) – Pre-op Evaluation, Lumpectomy with SN biopsy/Axillary dissection, Lumpectomy Re-excision, WBRT-B post lumpectomy (Konski), Mastectomy with SN biopsy/Axillary dissection, Breast Reconstruction	13,590.03	9,487.95–20,909.41

Bilateral Mammography (Screening)	49.76	33.23 – 73.65

Bilateral MRI	965.57	646.60 – 1,432.84

Unilateral Mammography	42.48	28.37 – 62.88

Unilateral MRI	711.72	476.51–1,055.97

Work Up – Ultrasound of Breast, Mammogram of One Breast, FNA Without Imaging, FNA With Imaging, Ultrasound-Guided Core Biopsy	591.10	435.49 – 832.66

Systemic Node Positive – CBC, CMP Office/Outpatient Visit New and Established, Heart First Pass (Single), Doxarubicin 60 mg/m2, Cyclophosphamide 600 mg/m2, Tamoxifen 180 tabs (Node Pos), Paclitaxel 175 mg/m2, Trastuzumab 4 mg/kg × 1 = 272 mg (2/3 vial over 90 minutes)	12,923.90	9,955.04–19,851.46

Mammogram BI-RADS 0/3 False Positive	42.48	28.37 – 62.88

MRI BI-RADS 0/3 False Positive	711.72	476.51–1,055.97

Refer to Additional File 1 for the current procedural terminology codes.

Abbreviations: APC, Ambulatory Payment Classification; BI-RADS, Breast Imaging Reporting and Data System classification; CBC, Complete Blood Count; CMP, Comprehensive Metabolic Panel; CPT, Current Procedural Terminology; DRG, Diagnosis Related Group; FNA, Fine Needle Aspiration; mg/kg, milligram per kilogram; MRI, Magnetic Resonance Imaging; SN, sentinel node; tabs, tablets; WBRT-B, whole breast external beam radiation therapy with a boost

### Utilities

Model utilities were measured according to quality adjusted life years (QALY) using values between 0 (death) and 1 (perfect) based on published literature. Measured utilities included the utility of having breast cancer, living, dying, having been diagnosed with node positive breast cancer, having experienced a false positive examination, having to experience screening, and having had a false negative node positive tumor. These utilities were applied to each health outcome in the model. As seen in the probabilities, ranges for sensitivity analyses were obtained by constructing 95% confidence intervals for proportions based on published work. Table 3 contains all base case probabilities and their respective ranges used in the sensitivity analyses. Neither costs nor outcomes were discounted since costs and benefits all occurred within the year that resources were utilized and each strategy required the recurring costs of screening. All outcomes were discounted at 5% per year, consistent with literature recommendations [23-25].

**Table 3:** Utilities and discount rate used in the model

**UTILITIES**	**BASE CASE**	**RANGE**
Breast Cancer	0.950	0.907 – 0.993
Alive	1.000	
Node Positive	0.800	0.722 – 0.878
Dead	0.000	
False Positive	0.890	0.829 – 0.951
Screening	0.990	0.970 – 1.000
False Negative Node Positive	0.660	0.567 – 0.753
Discount Rate	0.050	0.00 – 0.050

### Sensitivity Analyses

Univariate sensitivity analyses were performed on each individual cost, probability and utility in order to explore the effect that variation in model parameters can have on the incremental cost-effectiveness of the MRI strategy. Probabilities and utilities were varied over the ranges derived from their 95% confidence intervals. Variations in costs were based on estimated minimums and maximums from Medicare reimbursement data for hospital, physician, and laboratory services according to the methodology described in recently published work [11]. Costs for drugs were varied according to the minimum and maximum medication costs from the FSS. Both low and high incremental cost-effectiveness ratios (ICERs) were recorded in univariate analyses and parameters were varied across their distributions in probabilistic sensitivity analyses. Net health benefit assessments were performed using a $50,000/QALY willingness-to-pay threshold and alternative threshold values were examined. The values of individual model parameters above or below which MRI became cost-effective were recorded as thresholds. In addition to the univariate sensitivity analyses, a probabilistic sensitivity analysis was performed with 10,000 Monte Carlo simulations to assess the robustness of the findings in the base case. Confidence ranges for the incremental cost and effectiveness of both screening strategies were recorded. Normal distributions were used with base case values serving as the mean and standard deviations calculated from the high and low ranges for each parameter.

## Results

The MRI strategy provided 14.1 QALYs at a discounted cost of $18,167 while mammography provided 14.0 QALYs at a discounted cost of $4,760 over 25 years of screening (Table 4). The ICER of MRI compared to mammography was $179,599/QALY. Without discounting, MRI provided 23.6 QALYs at a cost of $30,380 compared to 23.4 QALYs for mammography at a cost of $7,765. Without adjustments for quality-of-life, MRI provided 23.9 life years (14.3 discounted life years) compared to 23.8 life years (14.2 discounted life years) for mammography producing a discounted ICER of $146,602/life year.

**Table 4:** Costs, quality-adjusted life years, cost-effectiveness ratio, and incremental cost-effectiveness ratio of the screening regimens over 25 years of screening

	Discounted*			**Undiscounted**		
**STRATEGY**	**COST ($)**	**QALYS**	**ICER ($)**	**COST ($)**	**QALYS**	**ICER ($)**

Mammography	4,760	14.0	---	7,765	23.4	---
MRI	18,167	14.1	179,599	30,380	23.6	124,291

Discounted at the rate of 5%

Abbreviations: CE, Cost-Effectiveness; ICER, Incremental Cost-Effectiveness Ratio; MRI, Magnetic Resonance Imaging; QALYs, Quality-Adjusted Life Years.

**Figure 2:**
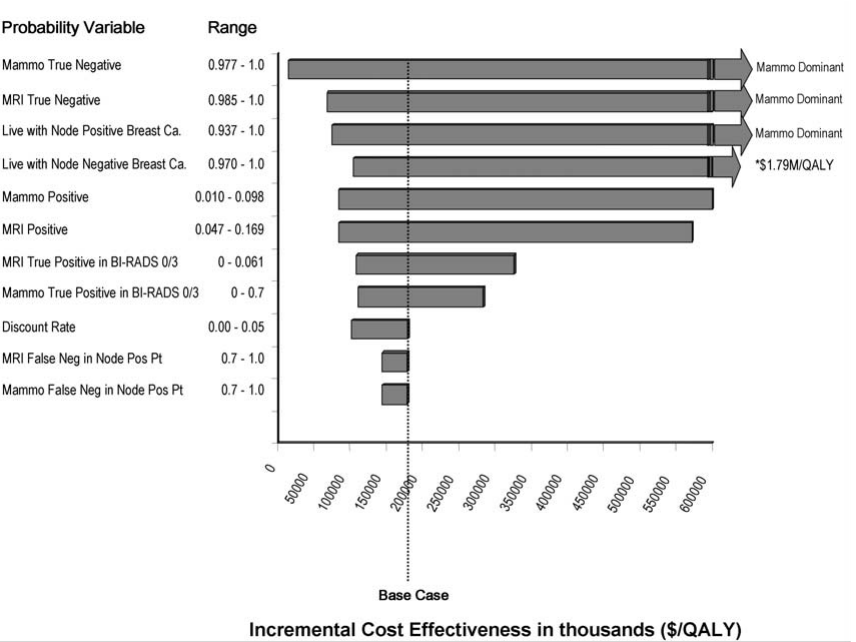
**Tornado diagram of univariate analyses**. This Tornado diagram shows the degree to which uncertainty in individual variables affects ICER.

In univariate analysis, breast MRI screening became < $50,000/QALY when the cost of the MRI was < $315. Univariate sensitivity analyses are displayed in a tornado diagram of the most influential variables (Figure 2). In this diagram, each bar represents the impact of uncertainty in an individual variable on the ICER. Model parameters that greatly influenced the ICER included the probability of living with a node negative cancer, and the probabilities associated with positive mammography and MRI readings. Additional File 2 provides the results for univariate analyses for all model parameters.

**Figure 3:**
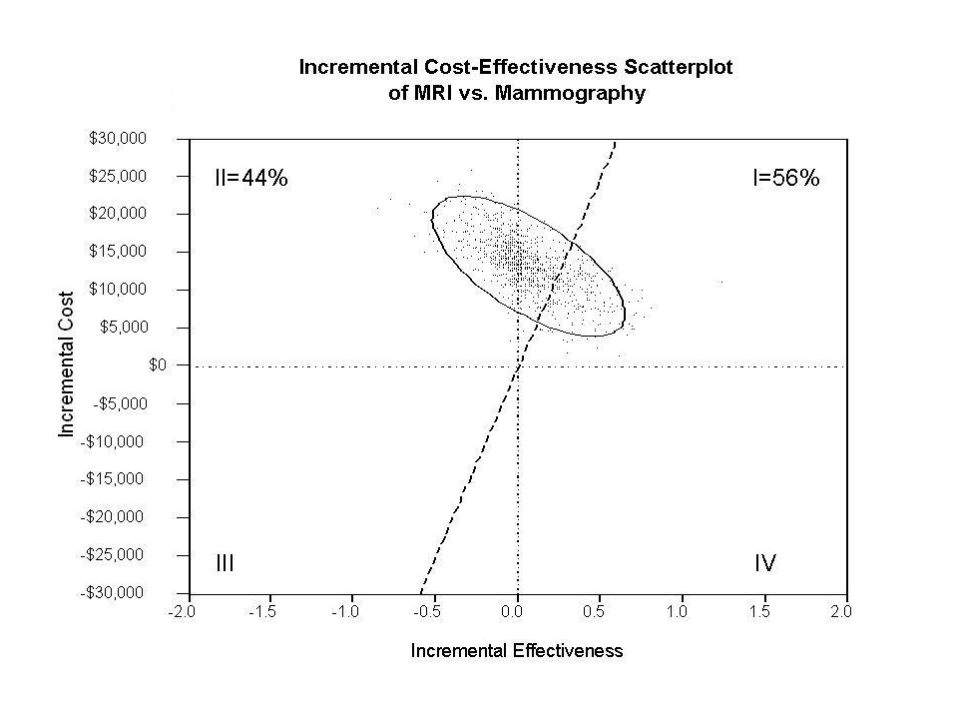
**Incremental cost and effectiveness of MRI over mammography**. This scatter plot shows the distribution of 10,000 trials form the Monte Carlo simulation.

**Figure 4:**
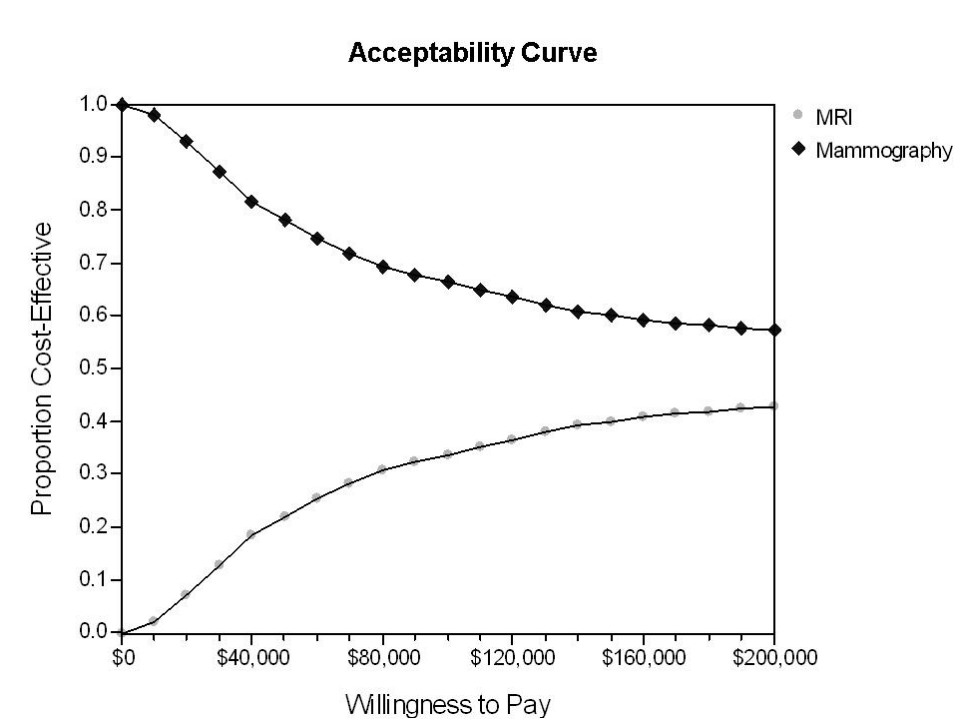
**Net health benefit acceptability curves**. This graph shows the proportion of trials that attained cost-effectiveness for a given strategy for willingness-to-pay thresholds up to $200,000/QALY.

In the probabilistic sensitivity analysis, MRI screening produced a net health benefit of -0.202 QALYs (95% central range: -0.767 QALYs to +0.439 QALYs) at a willingness-to-pay threshold of $50,000/QALY. The results of the probabilistic sensitivity analysis were plotted as an incremental cost-effectiveness scatterplot (Figure 3) to show the distribution of 10,000 trials from the Monte Carlo simulation. Each trial point provides a comparison of the incremental costs and benefits of MRI screening to mammogaphy. For each comparison, parameters for both screening strategies were simultaneously and randomly sampled from the probability, cost, and outcome distributions to account for uncertainty in the base case parameter estimates. The points could fall in four quadrants; Quadrant I, where the MRI screening strategy is both more costly and more effective than the standard regimen, contained 56% of the samples, 34% had an ICER of greater than $50,000/QALY and 22% had an ICER > $100,000/QALY. Quadrant II, where the MRI strategy is more costly but less effective (inferior), contained 44% of the samples. Quadrant III represents a situation where MRI screening is both less costly and less effective while Quadrant IV represents a situation where the MRI screening strategy is less costly and more effective (superior). Both Quadrants III and IV contained no points. The net health benefit acceptability curve shows the proportion of trials that attained cost-effectiveness for a given strategy for willingness-to-pay thresholds up to $200,000/QALY (Figure 4).

## Discussion and conclusion

Breast MRI may provide health benefits when compared to mammographic screening for some high-risk women; however, this approach does not appear to be cost-effective at a willingness-to-pay threshold of $50,000/QALY. This historical threshold is based on the cost of providing care to patients with end-stage renal disease in the 1970s, which now exceeds $120,000/QALY. Given the increased costs due to increased technology over this time period, and the benefits to be gained from the use of technological advancements, it follows that a higher threshold would be more appropriate and relevant. In this model, MRI screening does not approach cost-effectiveness even if a threshold of $120,000/QALY is used.

In this study, a series of univariate sensitivity analyses were conducted to explore the impact of varying all resource costs, probabilities, and utilities on the incremental cost-effectiveness of MRI screening. Our model showed that MRI screening became more cost-effective as the cost of MRI decreased and the cost of mammography increased. The cost-effectiveness of MRI screening in this model strongly depended on several factors, including the likelihood of survival with node-negative breast cancer, survival with node-positive breast cancer, positive mammography reading, and positive MRI reading. Therefore, the model suggests that screening with MRI becomes more cost-effective for patients with higher-risk profiles, and as the positive predictive value of MRI screening increases. Additionally, a probabilistic sensitivity analysis was performed to assess the robustness of the findings in the base case. The net health benefits of MRI screening relative to mammography improve as the willingness-to-pay threshold approaches $120,000/QALY, but even in this instance, it did not become cost effective for this population.

Other models have shown that MRI screening may be cost-effective in high-risk women, particularly those with BRCA1 and BRCA2 mutations. A study, by the UK Magnetic Resonance Imaging in Breast Screening Study Group, of 279 women at high familial risk for breast cancer found that the incremental cost per detected cancer in women with BRCA1 and BRCA2 mutations (n = 117) was £11,800 (2007 US $24,268) for contrast-enhanced MRI combined with mammography and £15,300 (2007 US $31,466) for contrast-enhanced MRI alone compared with mammography alone [11]. This study included women aged 35–49 years who tested positive or had a relative with BRCA1/BRCA2/TP53 mutation or had strong family history of breast/ovarian cancer. Also this study differs from our model in that, this study evaluated the cost effectiveness of MRI alone, mammography alone, and mammography in combination with MRI.

A recent cost-effectiveness analysis in the US by the Cancer Intervention and Surveillance Modeling Network consortium, in a simulated cohort of 25 year-old BRCA1 or BRCA2 mutation carriers born in 1980, found that using a threshold of $100,000/QALY gained resulted in MRI plus mammography screening being cost-effective from ages 35–54 in women with BRCA1 mutations ($89,661/QALY; the most cost-effective model in this group was $43,484/QALY for BRCA1 carriers ages 40–49), and for women with BRCA2 mutations < 50 years of age with extremely dense breasts on mammography ($98,454/QALY) [11,26]. Our study differs from this study in the patient cohort; we include women at high risk as per the Claus tables whereas this study only includes women with BRCA1/BRCA2 mutations. Also, this study evaluates the cost effectiveness of mammography alone compared to mammography plus MRI screening. In addition, the probabilities and utilities used in both the above studies are different from those used in our model.

Our study has some limitations that must be addressed. There are additional issues relevant to the management of women at high-risk for breast cancer that were not incorporated in the model, and may influence the cost-effectiveness of screening with MRI. For example, although BRCA mutation carriers may choose to undergo prophylactic mastectomy, many do not choose this option, with estimates ranging from 0% to 54% of carriers [11,26]. Furthermore, some of the women are also at increased risk for ovarian cancer. The costs of radiation exposure due to annual mammography starting at an earlier age were not incorporated, nor were the costs of possible anxiety and stress from unnecessary biopsies stemming from false positive MRI screening. Any or all of these factors might alter the cost-effectiveness estimate. Finally, the results of our model should be interpreted with care given that the results of this cost-effectiveness analysis require comparisons to data from observational studies, the Surveillance, Epidemiology and End Results Program, or clinical trials.

All probabilities and utilities used to populate the model are estimates derived from the literature. Each of these estimates carries inherent uncertainty, as does using a hypothetical cohort. Possible selection bias associated with utilizing the Claus tables may affect our base case effectiveness and resource use estimates by either over or underestimating our base case model parameters. Moreover, in our probabilistic sensitivity analysis, we did not assume that a correlation structure existed among the distributions of the parameters. However, both univariate and probabilistic sensitivity analyses were performed to address uncertainty in parameter estimates by exploring variability in each probability, cost, and outcome estimate.

Although the NCCN screening guidelines for women aged 25 years and older at high-risk for breast cancer include breast MRI as an adjunctive screening tool to mammograms, breast MRI has not yet been shown to decrease mortality. Further research into the appropriate role and cost-effectiveness of screening breast MRI will better elucidate which specific risk groups are more likely to benefit from MRI screening.

## Competing interests

The authors declare that they have no competing interests.

## Authors' contributions

SGM and CRF conceived the study, designed the research, and performed the statistical analysis. JWT and PJS designed the research, performed the statistical analysis, and drafted the manuscript. LCF performed the statistical analysis and drafted the manuscript. All authors read and approved the final manuscript.

## Pre-publication history

The pre-publication history for this paper can be accessed here:



## Supplementary Material

Additional file 1 **Current procedural terminology codes.** This table provides the current procedural terminology codes for procedures mentioned in Table 2. Click here for file

Additional file 2 **Results of univariate analyses.** This table provides the results of univariate analyses for all model parameters. Click here for file
